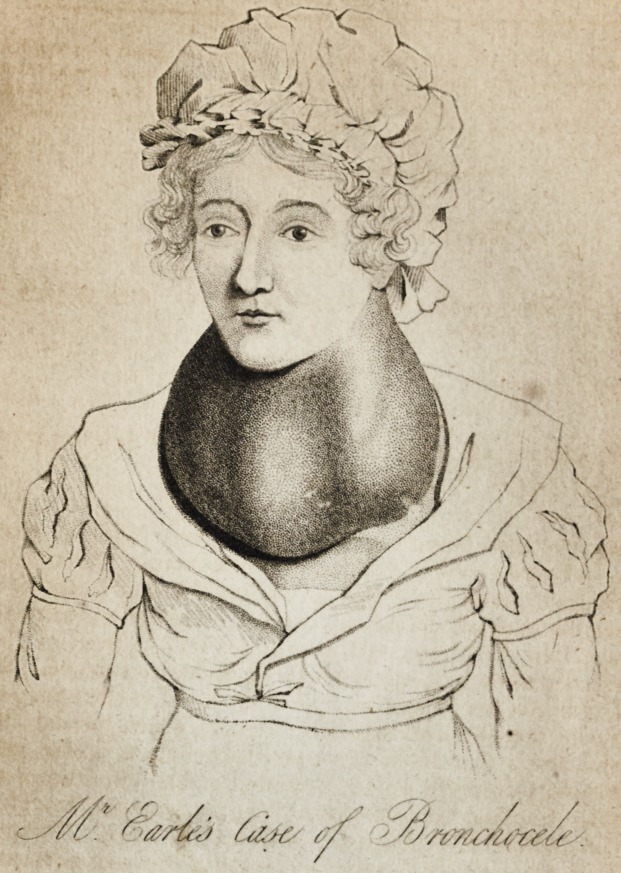# Case of Bronchocele, in Which the Superior Thyroid Arteries Were Tied

**Published:** 1826-09

**Authors:** H. Earle


Mr. Earle's Case of Bronchocele.

Mr. Chevalier's Case of Calculus.

Weight Six Drachms.

Pag 24

Engraved for the London Medical & Physical Journal. Sept 1 1826.

THE LONDON

### Medical and Physical Journal.

N° 331, VOL. LVI.] SEPTEMBER, 1826. [N° 3, *New Series.*

For many fortunate discoveries in medicine, and for the detection of numerous errors, the world is indebted to the rapid circulation of Monthly Journals; and there never existed any work, to which the Faculty, in Europe and America, were under deeper obligations, than to the Medical and Physical Journal of London, now forming a long, but an invaluable, series.—RUSH.

### ORIGINAL PAPERS.

### CASES OBTAINED FROM PUBLIC INSTITUTIONS, AND OTHER AUTHENTIC SOURCES.

### BRONCHOCELE.

*Case of Bronchocele, in which the superior Thyroid Arteries were tied.* By H. EARLE, F.R.S. &c.

[WITH AN ENGRAVING.]

JANE LARKING, aged seventeen years, a native of Malling, near Maidstone, labouring under a very large bronchocele, was admitted into St. BARTHOLOMEW'S HOSPITAL, on the 5th December, 1822, under the care of Mr. ABERNETHY. When about thirteen years of age, she first perceived a swelling on the front of the neck, which for some time caused little or no inconvenience. At the age of fourteen the catamenia appeared, and for the space of two years she menstruated regularly; during which period the tumor diminished. At sixteen menstruation became irregular, and the gland rapidly enlarged up to the period of her application at the hospital. She had for some years been occasionally troubled with pain in the chest, accompanied with severe cough. Various remedies were employed in the country, without the least success.

At the time of her admission, the gland was very painful, and had acquired considerable magnitude, causing great difficulty in respiration and deglutition. The carotid arteries were displaced from their natural situations, occupying the outer and posterior margins of the tumor. The superior thyroid arteries were observed much enlarged, their pulsation being evident at some distance. The state of her health was in every respect bad: menstruation irregular, bowels habitually costive, pulse rapid, tongue foul; she suffered much from pains in the head and drowsiness, and she complained of pain in her chest and cough.

The principal medical treatment employed consisted in endeavouring to improve the health, by regulation of diet and attention to the state of the secretions; the neck was bathed with tepid water. Under this plan of treatment some amendment took place; the tumor diminished rather in size, and was less painful.

After some time, her health appearing to suffer from confinement in the hospital, and the symptoms of affection of the lungs increasing, she was recommended to return into the country, and left the hospital about the middle of February.

On the 15th of July, 1823, she was again admitted into the hospital, under my care. She stated that, on her return into the country, her health improved and cough subsided, but the tumor continued to increase, particularly during the last two months, causing so much difficulty of respiration as at times to threaten suffocation. Finding herself daily getting worse, she determined to return to the hospital.

On the evening of her admission, her difficulty of breathing was extreme, and she was wholly incapable of swallowing any solid food; her pulse was 120; bowels costive; the catamenia had not appeared for five months. The cough and pain in the chest had returned, with aggravation; headache and drowsiness constant. The bronchocele was evidently much increased. The superior thyroid arteries were greatly enlarged; that on the right side communicating a peculiar thrill when felt, which led to the supposition of its coats being diseased.—Leeches and evaporating lotions were applied to the tumor; and Pil. Hydr. gr. v., and Pil. Aloës c. Myrrha gr. x., were ordered to be taken every night.

The leeches were repeated on the 16th, 19th, 22d, and 25th, without any decided benefit. She also took Liquor Potassæ Hydriod. m. x. ter die; but this producing considerable nausea, was soon discontinued.

On the 1st of August,* it was evident that the tumor had augmented since her admission, and her breathing was become so extremely laborious, that it was apparent she could not long survive without some relief being afforded. Under these circumstances, I determined on tying one of the thyroid arteries.

On the 2d, at half-past twelve A.M. I passed a ligature round the right superior thyroid, which was much the largest. The vessel was healthy, but enlarged to nearly the size of the carotid. The most acute pain in the head immediately followed the tightening of the ligature. The pulsation in the tracheal side of the artery diminished materially, but did not entirely subside. About half an hour after the operation, the pain in the head continuing, twenty ounces of blood were taken from the arm, which afforded some relief. Cold cloths were directed to be constantly applied to the tumor, and the patient's head was much elevated with pillows.

At four P.M. the pain had abated, but there was much drowsiness; the pulse at the wrist quick, but not full; the carotids

* The drawing, from which we have given a representation of the tumor, (see Frontispiece,) was taken this day.

beating with greater violence than before the operation, the pulsation being evident at some distance along the course of the thyroid up to the ligature.—Saline purgatives, with tincture of digitalis, were ordered for her.

August 3d.—She passed a bad night. Her pulse was rapid, tongue furred, and the drowsiness amounted to coma; the pulse in the carotids greatly exceeding in proportion that at the wrist. The bowels were moved with calomel and jalap, and twenty leeches were applied to the temples, which continued bleeding the whole day, and afforded great relief. In the evening, all her bad symptoms had abated, and her breathing was greatly relieved. She passed a good night, and continued to improve.

On the 6th, the tumor was measured, and found to be considerably smaller; respiration and deglutition performed with comparative facility; the pulsation in the thyroid and whole tumor much diminished: on the tracheal side it had ceased, and the left thyroid beat with much diminished force. The cough had nearly left her.

On the 11th, the neck was measured again, and the tumor was found to be much diminished. The portion of the artery between the ligature and the carotid had ceased to beat. The patient stated herself to breathe and swallow with greater ease than she had done for the last two years. The ligature came away in the evening in the poultice.

From this time to the 24th, every thing went on favourably. On measuring the neck, the tumor was found to have diminished three and a half inches in circumference, principally on the right side.

As the girl was anxious to return home to her friends, and her health began to suffer from confinement, she was dismissed on the 28th of August, with an understanding that, if the tumor at all encreased, she would return, and have the artery on the left side tied.

On the 11th of September she came back, in consequence of her finding the tumor remain stationary, and the artery on the left side pulsate with increased force. On the 17th, the artery was secured: it was healthy in its texture, and about the size of the radial artery. The patient suffered a good deal from hysteria and apprehension before the operation, dreading a return of the severe pain in the head and coma. Leeches had been previously applied, and her system much depleted by saline purgatives. No alarming symptoms followed the application of the ligature. The diminution in the size of the tumor was not nearly so great nor so rapid as after the first operation. The ligature came away on the 1st of October, on which day the menses reappeared, after an interval of nearly seven months.

On the 10th, the patient finally left the hospital, having no cough, no impediment in swallowing, and respiring with perfect freedom. The tumor was evidently smaller and softer, and appeared more divided in its texture. I heard from her in November, and again in January 1824, at which time the tumor continued slowly to decrease, and her health was greatly restored.

The fortunate termination of this case was the more gratifying, as it was the general opinion, before the first operation, that all attempts would be in vain, in consequence of the supposed diseased state of the girl's lungs.

---

### MALFORMATION OF THE RECTUM.

## Figures and Tables

**Figure f1:**